# Lymphomatoid granulomatosis associated with azathioprine therapy in Crohn disease

**DOI:** 10.1186/1471-230X-14-127

**Published:** 2014-07-14

**Authors:** William Connors, Cameron Griffiths, Jay Patel, Paul J Belletrutti

**Affiliations:** 1 Department of Medicine, University of Calgary, Calgary, Canada; 2 Department of Pathology, Foothills Medical Centre, Calgary, Canada; 3 Division of Gastroenterology and Hepatology, University of Calgary, Foothills Medical Centre, Calgary, Canada

**Keywords:** Lymphoproliferative disorders, Crohn disease, Inflammatory bowel disease, Azathioprine, Thiopurines

## Abstract

**Background:**

Lymphomatoid granulomatosis (LYG) is a rare Epstein-Barr virus-associated lymphoproliferative disorder. It most often occurs in patients with immunodeficiency and the clinical course ranges from indolent behavior to that of an aggressive malignancy. Pulmonary, central nervous system and dermatological manifestations are most common. To our knowledge this is the first reported case of LYG related to azathioprine therapy in Crohn disease.

**Case presentation:**

A twenty-six year old Caucasian woman with colonic Crohn disease on maintenance azathioprine therapy presented with right upper quadrant pain and fever. Diagnostic imaging revealed extensive liver, pulmonary and cerebral lesions. A diagnosis of LYG was made based on the pattern of organ involvement and the immunohistochemical features on liver and lung biopsy.

**Conclusions:**

Thiopurine therapy for inflammatory bowel disease is associated with an increased incidence of lymphoproliferative disorders. This report highlights the diagnostic challenges associated with LYG. As long-term thiopurine therapy remains central to the management of inflammatory bowel diseases it is essential that both patients and clinicians are aware of this potential adverse outcome.

## Background

Lymphomatoid granulomatosis (LYG), first described by Liebow *et al.* in 1972 [1], is a rare Epstein-Barr Virus (EBV)-associated lymphoproliferative disorder. LYG is classically described as primarily extranodal in nature involving the lungs, skin, central nervous system and liver. It is an incompletely understood clinical entity with a median survival of 14 months from the time of diagnosis. Age at first presentation is typically between the fourth and sixth decade with a male to female ratio of two to one. Although it may occur in immunocompetent hosts, there is a strong association with immune deficiency, both humoral and cell mediated. Clinical presentation is often non-specific with pulmonary involvement being most common and manifesting as cough, shortness of breath or chest pain [1-4].

Histopathologically, LYG is characterized as a mixed mononuclear cell infiltrate with atypical large CD20+ B-lymphocytes on the background of reactive CD3+ T- lymphocytes, histiocytes and plasma cells. The infiltrate is angiocentric and typically invades the microvasculature. It is graded from I to III based on both the number of large B-cells and the proportion that stain positively for EBV-encoded small RNA (EBER) by in situ hybridization (ISH) [2]. The presence of these EBER-positive large B-cells and vascular invasion are keys to the diagnosis of LYG. Grade I and II LYG are slowly progressive while grade III LYG is aggressive with poor prognosis and requires treatment with multi-agent immunochemotherapy.

Here we present a case of LYG in a 26-year-old Caucasian female receiving azathioprine maintenance therapy for Crohn disease (CD) who presented with primary gastrointestinal complaints and fever. This report will highlight the diagnostic challenges associated with LYG and describe what we believe to be the first documented case of this rare disease in the setting of azathioprine treatment for CD.

## Case presentation

A 26-year-old Caucasian woman presented with three days of right upper quadrant pain, fever, and malaise. She had a six-year history of Crohn colitis initially treated with high dose mesalamine (4 g daily) then transitioned to azathioprine (2.5 mg/kg) after loss of response. She had been in clinical remission on azathioprine for the preceding 14 months. The patient had not been on any other immune modulating or biologic therapies.

**Figure 1:**
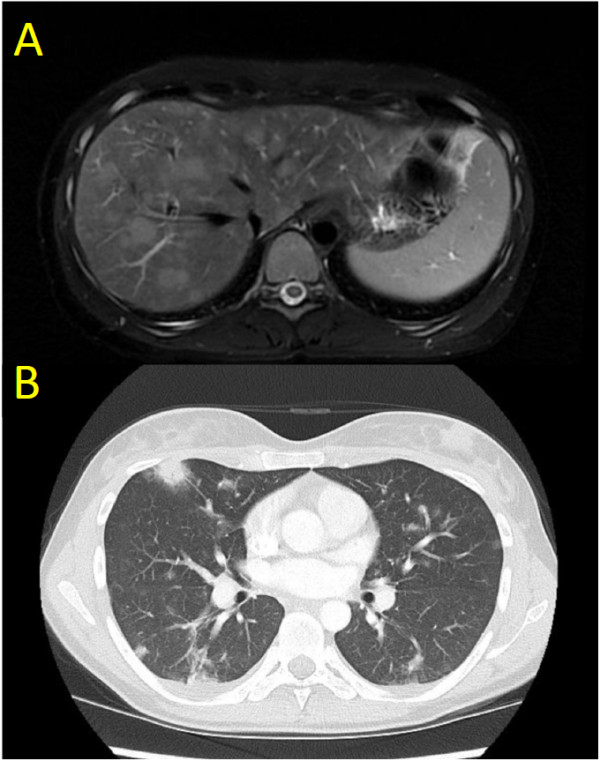
**Diagnostic imaging features of lymphomatoid granulomatosis in our case. A)** T2 weighted axial magnetic resonance (MR) image showing hyperintense lesions in the liver. **B)** CT axial image showing irregular bilateral pulmonary opacities with peripheral predominance and areas of ground-glass changes classic for lymphomatoid granulomatosis.

**Figure 2:**
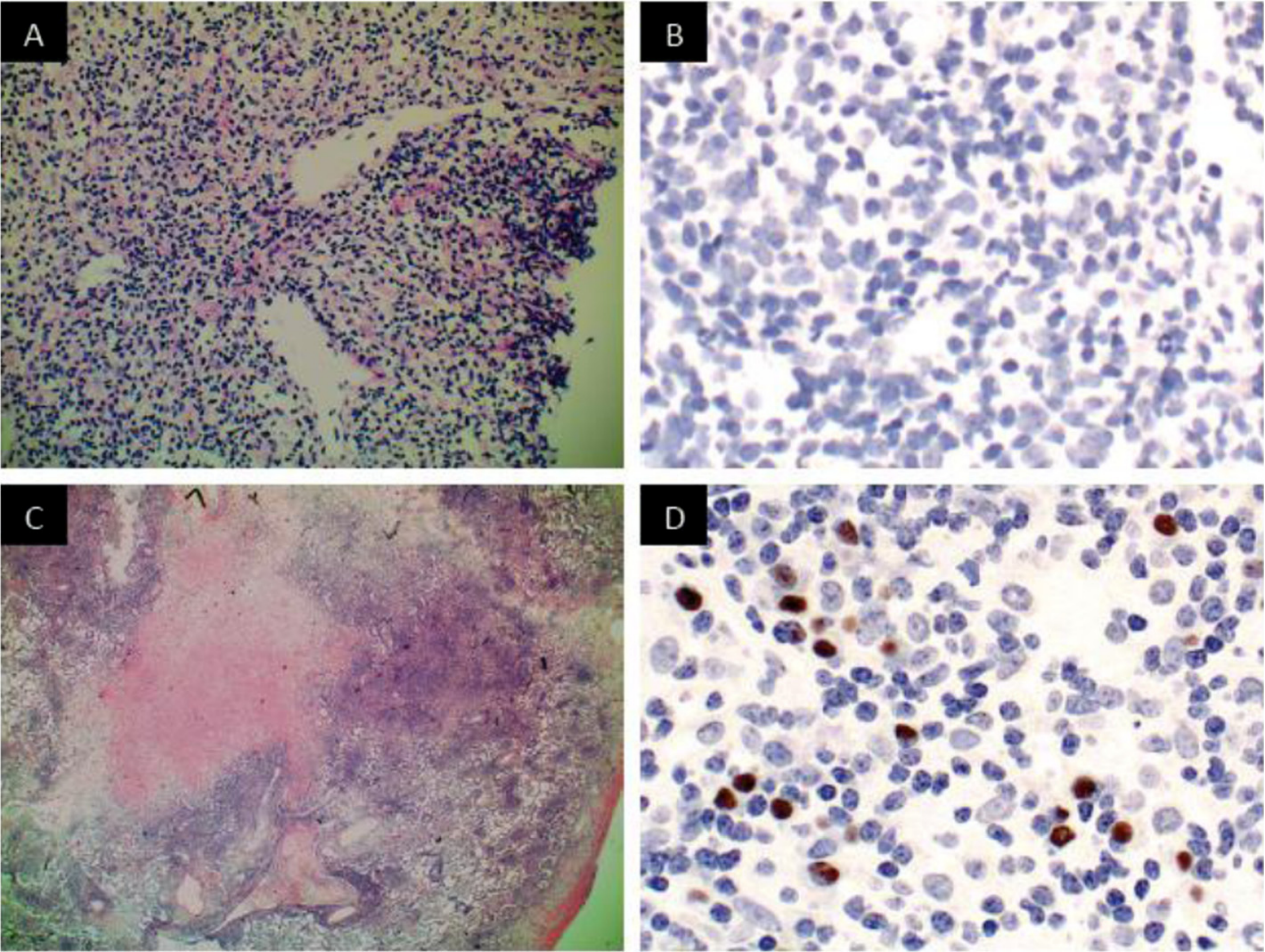
**Histopathology features of lymphomatoid granulomatosis in our case. A)** H&E stain of liver core biopsy with a periportal polymorphous infiltrate composed of small lymphocytes, histiocytes, and plasma cells. **B)** EBV in situ hybridization (EBER) of liver showing no positively stained cells. **C)** H&E stain of lung wedge biopsy showing an extensive, focally angiocentric, polymorphic infiltrate with geographic tissue necrosis. **D)** EBER of lung showing scattered positive nuclear staining in the distribution of B-cells (CD20 immunostain not shown).

**Figure 3:**
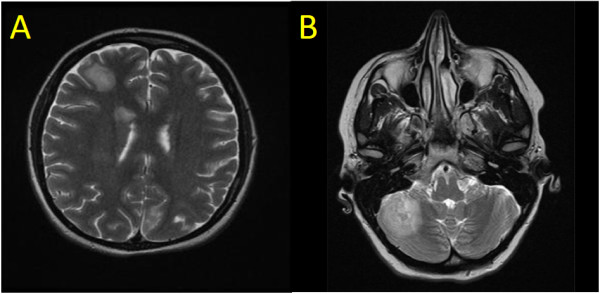
**Central nervous system lesion in our case.** T2 weighted MR image showing a large hyperintense lesion in **A)** right frontal cerebral cortex and **B)** right cerebellar cortex.

Physical examination revealed focal right upper quadrant abdominal tenderness but no palpable masses, organomegaly or signs of ascites. Laboratory review revealed new onset pancytopenia (hemoglobin = 119 g/L, neutrophils = 1.4 × 10^9^/L, platelets = 80 x10^9^/L), cholestatic liver enzyme elevation (GGT = 272 U/L, ALP = 342 U/L) and hepatic synthetic impairment (bilirubin = 19 umol/L, INR = 1.8, albumin = 24 g/L). Azathioprine therapy was suspended. Investigations failed to identify a source of infection; however, magnetic resonance (MR) imaging revealed hepatomegaly with periportal, gastrohepatic, and retroperitoneal lymphadenopathy. A clinical diagnosis of azathioprine-associated pancytopenia with a concurrent non-A-E viral hepatitis was favored. She was administered G-CSF and transitioned to oral antibiotics. After clinical improvement, the patient was discharged from hospital with planned repeat MR imaging in one to two months.Six weeks later, she developed progressive abdominal pain, night sweats, and non-positional presyncopal symptoms. The patient did not report diarrhea or GI bleeding. Endoscopic evaluation of the upper GI tract and colon were normal with no evidence of inflammation or active Crohn disease. She had also developed her first outbreak of genital herpes (serologically HSV-2) for which she was started on antiviral therapy. Repeat MR imaging now demonstrated extensive T2 hyperintense lesions distributed throughout her liver and both kidneys (Figure 1A).Over the next weeks in hospital, she experienced progressive abdominal bloating, malaise, dyspnea and night sweats without documented fevers. Computed tomography (CT) of her lungs revealed extensive, peripherally distributed, bilateral ground-glass opacifications (Figure 1B). Bronchoalveolar lavage and transbronchial biopsy were negative for infection or malignancy. A comprehensive infectious diseases work-up was negative; of note serology and PCR viral titers for EBV were negative. HIV serology was negative. Core biopsy of the liver revealed atypical B-lymphocyte proliferation with prominent angiocentricity (Figure 2A). The EBV encoded small RNA (EBER) staining by in situ hybridization was negative (Figure 2B). Thus the liver biopsy was classified by hematopathologist consensus as an “atypical lymphoid infiltrate”. Further work-up with a bone marrow biopsy showed no morphologic abnormalities and was EBER-negative. An MR of the head revealed multiple cerebellar, cortical and subcortical enhancing lesions (Figure 3). As the lesions appeared to be infarcts of different ages across different territories, a vasculitic process was suggested but a subsequent serological work-up was negative.

The patient opted to leave hospital pending consultation opinions on her liver biopsy and one month later returned to hospital with worsening dyspnea, cough and fevers. She underwent video-assisted thoracoscopic surgery (VATS) during which a wedge biopsy of her lung was obtained. In the days prior to surgery she had developed right pleural effusion and lower lobe pneumonia. Cultures from her pleural fluid grew Streptococcus milleri and Prevotella species, therefore she received a six-week course of parenteral antibiotics.Now four months after her initial presentation, the VATS-acquired lung wedge biopsy demonstrated EBER positivity in an atypical CD20+ lymphocyte infiltrate with vascular invasion sufficient to confirm a diagnosis of grade II LYG (Figure 2C, D). Expert pathology opinion from the National Institutes of Health (Bethesda, MD) concurred with the diagnosis. Over this period the patient had several transient episodes of left lower face and tongue paresthesias and repeat brain MRI revealed interval development of new enhancing lesions in both cerebral hemispheres with mild surrounding edema and small internal foci of microhemorrhage.

Treatment was initiated with subcutaneous recombinant interferon-alpha three times weekly titrated up to 10 million units per m^2^ of body surface area and four weekly doses of rituximab 375 mg/m^2^. Her treatment course was complicated by neutropenia requiring granulocyte colony stimulating factor (G-CSF), choledocholithiasis requiring cholecystectomy and admission to hospital with recurrent pneumonia. With therapy, all of her systemic lesions have steadily improved. This patient is alive and tolerating ongoing therapy at 12 months follow-up.

Our patient is the first reported case we are aware of with LYG in the context of CD and azathioprine use. Her presentation is atypical for LYG due to her young age, female gender, and the predominance of hepatic involvement preceding other pulmonary and CNS manifestations. In the larger context of LYG, only three percent of cases present with gastrointestinal symptoms; the majority primarily manifest pulmonary symptoms [1-4].

In our case, the diagnosis of LYG was suspected based on pattern recognition on lung imaging and suggestive liver pathology. The negative EBV staining and lack of necrosis on the liver biopsy sample pointed away from LYG as the diagnosis. After open lung biopsy, however, the classic features of LYG were revealed, namely atypical large B-cells that were EBER-positive, vascular invasion and necrosis. These pathological findings along with the patient’s clinical presentation and pattern of organ involvement clinched the diagnosis of LYG. This case highlights the difficulty of establishing the diagnosis of LYG in many cases due to sampling error and histological heterogeneity in a given patient depending on the site biopsied [5].

The current literature on LYG in association with CD or azathioprine is limited to two case reports. The first was a 17-year-old female patient with CD who developed LYG (stage III) with nasopharyngeal and pulmonary predominance following six years of 6-mercaptopurine therapy. She received systemic immunochemotherapy and was in clinical remission at four years follow-up [6]. The second was a 69-year-old female who developed LYG (stage III) with central nervous system predominance following ten months of azathioprine therapy for autoimmune hepatitis. Despite initiation of systemic therapy the patient died 34 days after diagnosis due to sepsis and cardio-pulmonary failure [7]. From these two case reports an independent association between the two major features of our case -CD and azathioprine (a thiopurine pro-drug of 6-mercaptopurine)– with the development of LYG can be theorized.

Multiple population-based studies of inflammatory bowel diseases (IBD) have not shown an excess risk of lymphoproliferative disorders (LD) over baseline in part because it is difficult to rule out an effect from the disease itself in the population of patients who require more intensive therapy [8,9]. However, studies evaluating the risk of LD in the setting of CD and thiopurine therapy, summarized in a review by Beaugerie *et al*[9], support an approximate five-fold therapy specific elevated risk. Along with other smaller retrospective cohort analyses, the CESAME study, a nationwide French prospective observational cohort study of 19,486 patients with IBD, showed an independent association between ongoing thiopurine therapy for IBD and the risk of LD (multivariate-adjusted hazard ratio = 5.28, CI 2.01-13.9, p = 0.0007) [10-12]. It should be noted that the CESAME Study Group concluded the absolute cumulative risk of LD after ten years of thiopurines was less than one percent. This cohort had no patients diagnosed with LYG.

Consistent with the findings in our patient, a comprehensive review of all LYG case series by Katzenstein *et al.*[4] illustrates the central role of underlying primary or secondary immunodeficiency leading to EBV reactivation. This may result in viral binding of lymphocyte membrane proteins and subsequent viral genome insertion facilitating oncogenesis [4]. Iatrogenic T-cell immunosuppression secondary to thiopurines is consistent with this proposed pathogenesis and was likely the predisposing factor in our case. Crohn disease-related lymphomas have been shown to have a higher prevalence of EBV-related subtypes, and in some EBV-related lymphoproliferative disorders disease remission has been seen with withdrawal of immunosuppression [13].

## Conclusions

Here we present the first case in the literature of lymphomatoid granulomatosis in the setting of azathioprine therapy for Crohn disease. LYG is one of several EBV-driven conditions that should be considered when a lymphoproliferative disorder develops in an immunocompromised patient.

The atypical features in our case underscore the importance of maintaining a high degree of clinical suspicion and diagnostic vigilance when evaluating non-specific abdominal symptoms frequently encountered in CD patients. This case highlights that even limited exposure to such therapy may induce LYG. Future research is needed into the pathogenesis and early detection of this disease to allow both clinicians and patients to make informed decisions and to raise awareness of this uncommon disorder.

## Consent

Written informed consent was obtained from the patient for publication of this Case report and any accompanying images. A copy of the written consent is available for review by the Editor of this journal.

## Abbreviations

ALP: Alkaline phosphatase; CD: Crohn disease; CNS: Central nervous system; CT: Computed tomography; EBV: Epstein-Barr virus; EBER: Epstein-Barr virus encoded small RNA; GGT: Gamma glutamyl transferase; IBD: Inflammatory bowel disease; ISH: In situ hybridization; LD: Lymphoproliferative disorder; LYG: Lymphomatoid granulomatosis; MR: Magnetic resonance.

## Competing interests

The authors declare that they have no competing interests.

## Authors’ contributions

WC and CG wrote the manuscript and performed the literature review. JP and PJB conceived of the report, participated in expert review and editing of the final manuscript. All authors read and approved the final manuscript.

## Pre-publication history

The pre-publication history for this paper can be accessed here:

http://www.biomedcentral.com/1471-230X/14/127/prepub
